# A synaptic mechanism for network synchrony

**DOI:** 10.3389/fncel.2014.00290

**Published:** 2014-09-18

**Authors:** Simon T. Alford, Michael H. Alpert

**Affiliations:** Department of Biological Sciences, University of Illinois at ChicagoChicago, IL, USA

**Keywords:** lamprey, oscillation, SK2, K_Ca_2, NMDA, locomotion, calcium, dendrites

## Abstract

Within neural networks, synchronization of activity is dependent upon the synaptic connectivity of embedded microcircuits and the intrinsic membrane properties of their constituent neurons. Synaptic integration, dendritic Ca^2+^ signaling, and non-linear interactions are crucial cellular attributes that dictate single neuron computation, but their roles promoting synchrony and the generation of network oscillations are not well understood, especially within the context of a defined behavior. In this regard, the lamprey spinal central pattern generator (CPG) stands out as a well-characterized, conserved vertebrate model of a neural network (Smith et al., 2013a), which produces synchronized oscillations in which neural elements from the systems to cellular level that control rhythmic locomotion have been determined. We review the current evidence for the synaptic basis of oscillation generation with a particular emphasis on the linkage between synaptic communication and its cellular coupling to membrane processes that control oscillatory behavior of neurons within the locomotor network. We seek to relate dendritic function found in many vertebrate systems to the accessible lamprey central nervous system in which the relationship between neural network activity and behavior is well understood. This enables us to address how Ca^2+^ signaling in spinal neuron dendrites orchestrate oscillations that drive network behavior.

## Introduction

Orchestration of neuronal activity within networks is integral to correct execution of behavior. Synchronization between groups of neurons is an organizational feature of many neural networks found in the central nervous systems of invertebrates (Wehr and Laurent, 1996; Riffell et al., 2009) to vertebrates (Womelsdorf et al., 2014) alike, and between microcircuits. Large-scale synchrony between neurons is particularly evident in the spinal (Grillner, 2003; Goulding, 2009) and brainstem networks (Koshiya and Smith, 1999) controlling rhythmic movement, but are also common to hippocampal and neocortical networks (Buzsáki and Draguhn, 2004; Grillner et al., 2005; Yuste et al., 2005). Synchronously active microcircuits, like the neurons that comprise the lamprey spinal central pattern generator (CPG), are driven through the synaptic connectivity of excitatory and inhibitory neurons combined with intrinsic burst-terminating electrical properties (Wallén and Grillner, 1987; Buchanan, 1993). However, little is known about the electrical and integrative properties of the complex dendritic architecture of lamprey spinal neurons where synaptic- and voltage-dependent conductances shape potentials arriving at the soma. In contrast, the integrative properties of cortical pyramidal neuron dendrites and their synaptic inputs have been extensively characterized (Spruston, 2008), while less is known about how these intrinsic properties generate rhythmic network activity, and ultimately the behaviors they are thought to subserve. To understand how neural networks generate complex patterns of activity underlying behaviors, it will be necessary to understand both the specific patterns of connectivity between neurons and how individual neurons respond to the inputs that they receive. Thus, this review seeks to merge disparate fields of research—dendritic integration and spinal central pattern generation. In doing so, we hypothesize that the ionic mechanisms driven through two rhythm-generating conductances, namely the synaptic interaction between ensembles of NMDA receptors (NMDARs) and Ca^2+^-dependent K^+^ channels, may have general implications for the synchronization of spinal to cortical networks. Thus, to explore the idea that active dendritic properties are at the core of this behavior, we examine in detail the lamprey spinal network and draw from other areas of dendritic research to enhance our understanding of what occurs at the level of the dendritic synapse to generate behavior.

## Supraspinal networks in the brainstem initiate and maintain locomotor drive

**Figure 1:**
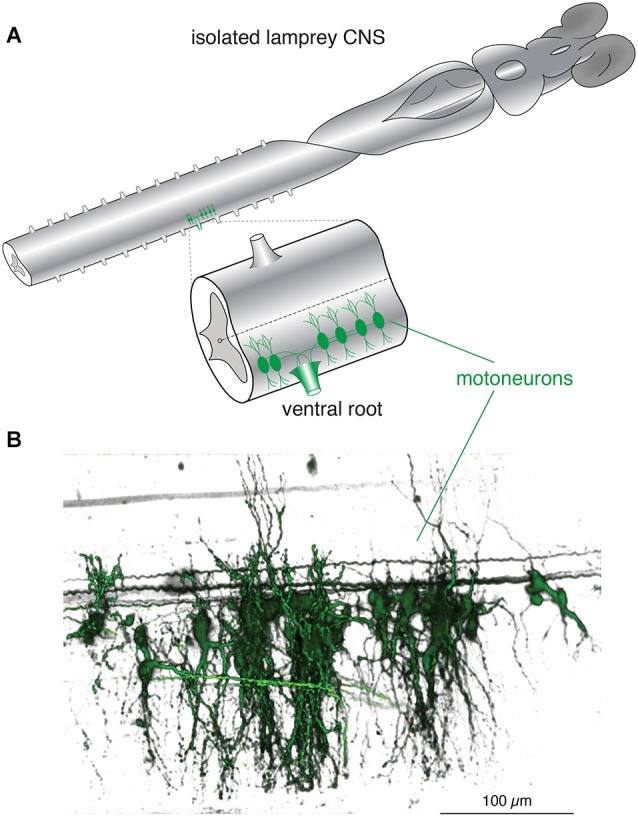
**Lamprey spinal motoneurons have a complex dendritic architecture. (A)** Schematic representation of an isolated lamprey brain and spinal cord. Spinal motoneurons and their complete dendritic architecture can be retrogradely labeled through an intramuscular injection of a dextran-conjugated fluorescent dye. Labeling (green) is visible on the side and segment of injection through axons converging into ventral roots (VRs) and to their respective neurons. Expansion shows a single spinal segment with multiple motoneurons labeled as in **(B)**. **(B)** A 3D reconstruction of motoneurons labeled from one spinal ventral root to emphasize the complexity of their structure and dendritic trees. Neurons were labeled by injecting the muscle wall of an animal with 5 μL of 5 mM Oregon Green 488 BAPTA1 Dextran. After 24 h the animal was sacrificed and the spinal cord fixed and cleared. A confocal stack was imaged to generate the full extent of the motoneurons in one spinal segment (Viana di Prisco and Alford, 2004).

Vertebrate locomotion is initiated and maintained by evolutionarily conserved serial pathways originating in the forebrain (Ericsson et al., 2013; Grillner et al., 2013), projecting to the mesencephalic locomotor region (MLR; Dubuc et al., 2008) and then to command neurons of the reticulospinal (RS) system, which innervates the entire rostro-caudal extent of the spinal cord, including cervical and lumbar centers in mammals (Goulding, 2009), and all segmental levels in fish as well as lamprey (Buchanan et al., 1987). However, following their activation by the brainstem, it is the circuits and neurons of the spinal CPG (Buchanan and Cohen, 1982) that create the complex synergy that rhythmically activates the locomotor musculature (Grillner et al., 2008). The structure of descending commands to spinal CPGs and the synaptic connectivity of the spinal network itself provides an opportunity to understand how dendritic activation within behaviorally relevant circuits underlies the astonishing complexity of vertebrate behavioral patterns. The circuitry of the lamprey CPG is well understood (Grillner et al., 2000, 2008) including the identities of the key neurons (Rovainen, 1974; Buchanan and Cohen, 1982), their neuronal targets, and neuropharmacology (Alford et al., 2003). However, in common with most neurons, these circuit components possess a complex dendritic morphology (Figure 1), yet we understand little of the spatiotemporal profile of dendritic activation within these neurons and the role that such patterns of activation might play in the physiological activity of the neurons during behavior. This lack of understanding is true for simple inputs, but particularly during goal-directed locomotion. This is partly because tracing the spatial distribution of physiological targets of neurons is challenging, but also because most studies of CPGs, whether in simple systems like the lamprey, or in more complex systems such as mammals, use isolated spinal cords and activate the networks pharmacologically (Sigvardt et al., 1985; Rossignol et al., 1998; Kyriakatos et al., 2011). This undoubtedly obscures the precise physiologically relevant spatiotemporal activation patterns of dendritic synapses that would otherwise drive these behaviors *in vivo*. In studies of spinal motor activity this has been largely overlooked perhaps due to the strong resemblance of electrophysiological output (i.e., fictive locomotion), or even actual movement, to observed locomoting animals. Despite this similarity, it is crucial to understand how physiological patterns of synaptic input and intrinsic membrane electrodynamics generate rhythmic behaviors.

## The synaptic connectivity of the spinal CPG network drives rhythmic network oscillations

**Figure 2:**
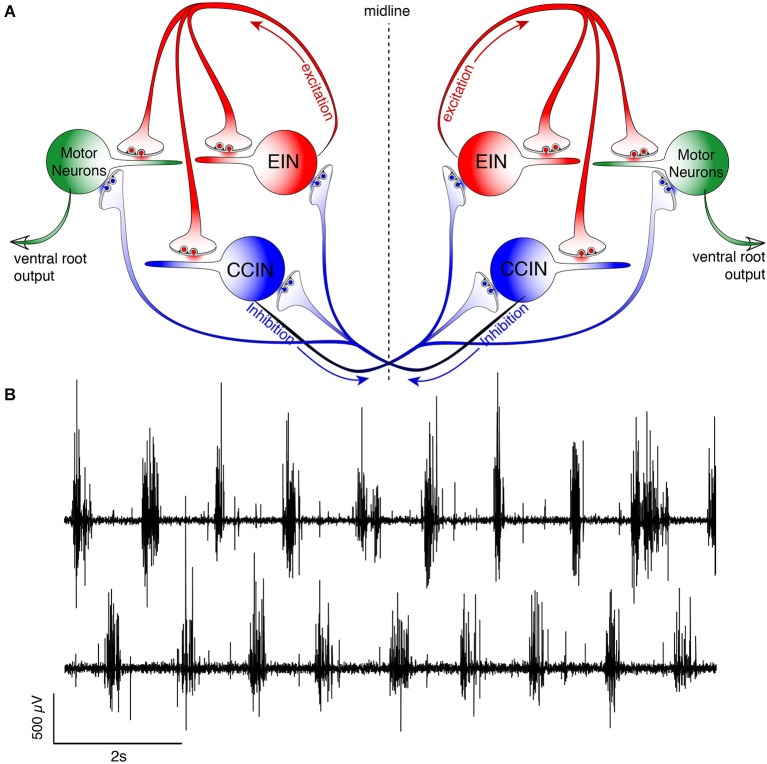
**Schematic representation of the lamprey spinal central pattern generator. (A)** Spinal CPG neurons receive both ipsilateral glutamatergic (red) input from excitatory interneurons (EINs, red) and contralaterally projecting glycinergic inhibition (blue) from reciprocally inhibiting, crossed glycinergic interneurons (CCINs, blue). Output of the CPG occurs from motoneurons (green), which directly synapse onto myotomal cells of the trunk musculature to cause muscle contraction producing rhythmic locomotion. **(B)** Output pattern recorded using glass suction electrodes from paired, contralateral (top vs. bottom traces) VRs showing alternating bursting of the spinal network during rhythmic locomotion. The reciprocally connected network described in **(A)** prevents excitation of the contralateral spinal cord when the ipsilateral side is bursting for each cycle (burst-to-burst), leading each side of the spinal cord to be precisely 180° out-of-phase from the other (Alford et al., 2003).

The very fluid, controlled nature of lamprey locomotion is produced after RS axons activate the local circuit neurons within the spinal ventral horn (Figure 2). Among these neurons, collectively referred to as ventral horn neurons (VHNs), the best characterized neurons responsible for pattern generation are excitatory interneurons (EINs) that provide ipsilateral, glutamatergic excitation (Buchanan and Grillner, 1987; Buchanan et al., 1989), while crossed caudally projecting interneurons (CCINs) provide contralateral, glycinergic inhibition (Grillner and Wallén, 1980; Alford and Williams, 1989). Motor neurons are the final common output neuron of each segment, which bundle into ventral roots (VRs) as they leave the spinal cord, before synapsing directly onto myotomal cells of the trunk musculature (Buchanan and Cohen, 1982). The precise, synaptic connectivity of the VHNs within and between individual segments serves to ipsilaterally excite (i.e., EINs), while simultaneously delivering contralateral inhibition (i.e., CCINs; Buchanan and Grillner, 1987). This reciprocally inhibited network ensures that within each segment, when one side of the trunk musculature contracts, the contralateral side is inhibited. Lateral interneurons, which project ipsilaterally to inhibit CCINs, facilitate the relief of reciprocal inhibition (Buchanan, 1982). However, the importance of lateral interneurons in maintaining network rhythmicity has been less emphasized because alternating, rhythmic bursting can persist in their absence as demonstrated by computer simulation (Wallén et al., 1992).

Work in lamprey (Grillner et al., 1981; Brodin et al., 1985, 1988; Brodin and Grillner, 1985; Buchanan and Grillner, 1987), *Xenopus* tadpoles (Dale and Roberts, 1984; Roberts and Alford, 1986), rats (Kudo and Yamada, 1987), and cats (Douglas et al., 1993) demonstrates that spinal glutamate receptor-mediated transmission activates and maintains locomotion. These data are supported by recordings of excitatory postsynaptic potentials (EPSPs) onto motoneurons and premotor interneurons (Dale and Roberts, 1985; Brodin et al., 1988; Noga et al., 2003) and pharmacological manipulation of the resultant behaviors (Brodin and Grillner, 1985; Dale and Roberts, 1985; Cazalets et al., 1992; Chau et al., 2002; Rybak et al., 2006). This neurotransmission both directly excites neurons of the CPG, and also activates complex non-linear membrane interactions, or oscillations, in these neurons mediated by NMDAR voltage-dependency and Ca^2+^ permeability coupled to the activation of Ca^2+^-dependent currents. The cellular processes underlying such oscillations are believed to be central to the coordination of locomotor behavior. In lampreys the identity of the descending glutamatergic RS command neurons is well-defined (Dubuc et al., 2008) and similarly spinal neurons that release glutamate locally within the spinal ventral horn (i.e., EINs) have been identified (Buchanan et al., 1989; Buchanan, 1993) as has their network role (Wallén et al., 1992).

One prominent feature of the spinal network is that it transforms unpatterned, exogenous glutamatergic input into a patterned, rhythmic output. The details of synaptic connectivity responsible for this phenomenon have been substantially explored in the lamprey (Wallén and Grillner, 1987; Grillner et al., 2001; Grillner, 2006) and the *Xenopus* embryo (Dale and Roberts, 1984, 1985). More recently, work in higher vertebrates (Masino et al., 2012) has emphasized how well conserved this network motif is throughout the vertebrate subphylum including lampreys, fishes, amphibians, chelonids and mammals (Dale and Roberts, 1984; Sigvardt et al., 1985; Kudo and Yamada, 1987; Hernandez et al., 1991; Guertin and Hounsgaard, 1998; Gabriel et al., 2009; Masino et al., 2012). After complete spinal transection (Cohen and Wallén, 1980; Brodin et al., 1985), the lamprey swimming network can still generate the electrophysiological correlates of swimming. While recording output from pairs of contralateral VRs using glass suction electrodes, excitatory amino acid (EAA) receptor agonists, such as kainate, *D*-glutamate, or *N*-methyl-*D*-aspartic acid (NMDA; Brodin et al., 1985; Brodin and Grillner, 1985; Wallén and Grillner, 1987), bath-applied to an isolated spinal cord (devoid of muscle or any other surrounding tissue) generates antiphasic bursts of activity across the spinal midline—the phase relationship across sides of the spinal cord is enforced by glycinergic inhibition (Cohen and Wallén, 1980; Alford and Williams, 1989)—and the same rostro-caudal phase lag as seen in intact behavior (Wallén and Williams, 1984). This network behavior, termed “fictive locomotion”, refers to the electrical output of the spinal CPG. Thus, the network acts as a CPG, a term that refers collectively to centrally located, local circuit spinal neurons that provide precise rhythmic output from spinal motoneurons. The spinal CPG operates in the absence of both sensory feedback from the spinal dorsal roots or descending networks and is found in all vertebrates (Kahn and Roberts, 1978; Forssberg et al., 1980; Roberts et al., 1981; Sholomenko and Steeves, 1987; Delvolvé et al., 1997; Field and Stein, 1997; Masino and Fetcho, 2005). Thus, the ability to generate rhythmic output via network oscillations is inherent to the spinal network itself and does not require supraspinal control.

## Single neurons are intrinsically rhythmic

The study of spinal neurons offers a unique insight into how properties of neural networks emerge from membrane activity at the cellular level and provides a straightforward behavioral context—locomotion—in which to place this activity. EAA agonists, like NMDA, cause the membrane potential (*V_m_*) of individual VHNs in isolated spinal cords to undergo repetitive oscillations that are in-phase with the ipsilateral VR of the corresponding hemi-segment (Sigvardt et al., 1985; Wallén and Grillner, 1987). During the depolarized phase, the cells can fire multiple action potentials (APs) before the cell is repolarized. This finding demonstrates how electrical properties of single cells within a network scale to direct the behavior of the network at large. Most VHNs oscillate in NMDA driven by phase-appropriate synaptic excitation from EINs and subsequent hyperpolarization from CCINs (Buchanan and Cohen, 1982). However, when tetrodotoxin (TTX) is applied, spiking is abolished, while the underlying *V_m_* oscillation persists (Wallén and Grillner, 1987). Since TTX pharmacologically isolates the recorded neuron by preventing synaptic communication within the network, the cell then oscillates with tonic exposure to NMDA. This phenomenon, termed NMDA-dependent, TTX-resistant oscillations (NMDA-TTX oscillations), is seen in most lamprey VHNs. This demonstrates that spinal neurons show intrinsic membrane properties that are capable of hyperpolarizing the cell during constant depolarizing challenge from an agonist. The net effect is to produce *V*_m_ oscillations. Removal of Mg^2+^ from the perfusing Ringer’s solution abolishes the oscillation and causes the neurons to remain at depolarized potentials because Mg^2+^ confers voltage-sensitivity to the NMDAR (Wallén and Grillner, 1987). Thus, the intrinsic membrane property of spinal neurons that causes oscillations is subject to the voltage-dependency of Mg^2+^ block of the NMDAR.

More generally within the nervous system, NMDARs have been well characterized as non-specific cation channels permeable to Na^+^_,_ K^+^, and Ca^2+^ (MacDermott et al., 1986; Ascher and Nowak, 1988). More recently, NMDAR-dependent Ca^2+^ entry has been demonstrated to be integral to dendritic computation (Branco et al., 2010) through regenerative “NMDA spikes” in pyramidal neurons (Schiller and Schiller, 2001; Larkum et al., 2009) with roles spanning from the induction of synaptic plasticity (Alford et al., 1993) to behavior (Smith et al., 2013b). In lamprey VHNs, removal of Ca^2+^ from the ringer and replacement with Ba^2+^ (an equivalent divalent cation which can also permeate Ca^2+^ ionophores) during NMDA-TTX oscillations causes the cell to become similarly trapped at a depolarized *V_m_*. Thus, Ca^2+^ is necessary to hyperpolarize the cell from the depolarized state. Ca^2+^ activates myriad Ca^2+^-dependent proteins. In particular, VHNs contain Ca^2+^-dependent K^+^ channels (El Manira et al., 1994; Wall and Dale, 1995; Han et al., 2007; Li and Bennett, 2007), which upon binding Ca^2+^, rapidly open a K^+^ channel that hyperpolarizes the cell. This “excitation-inhibition coupling” is a mechanism that effectively allows the cell to “turn off” autonomously following activation.

**Figure 3:**
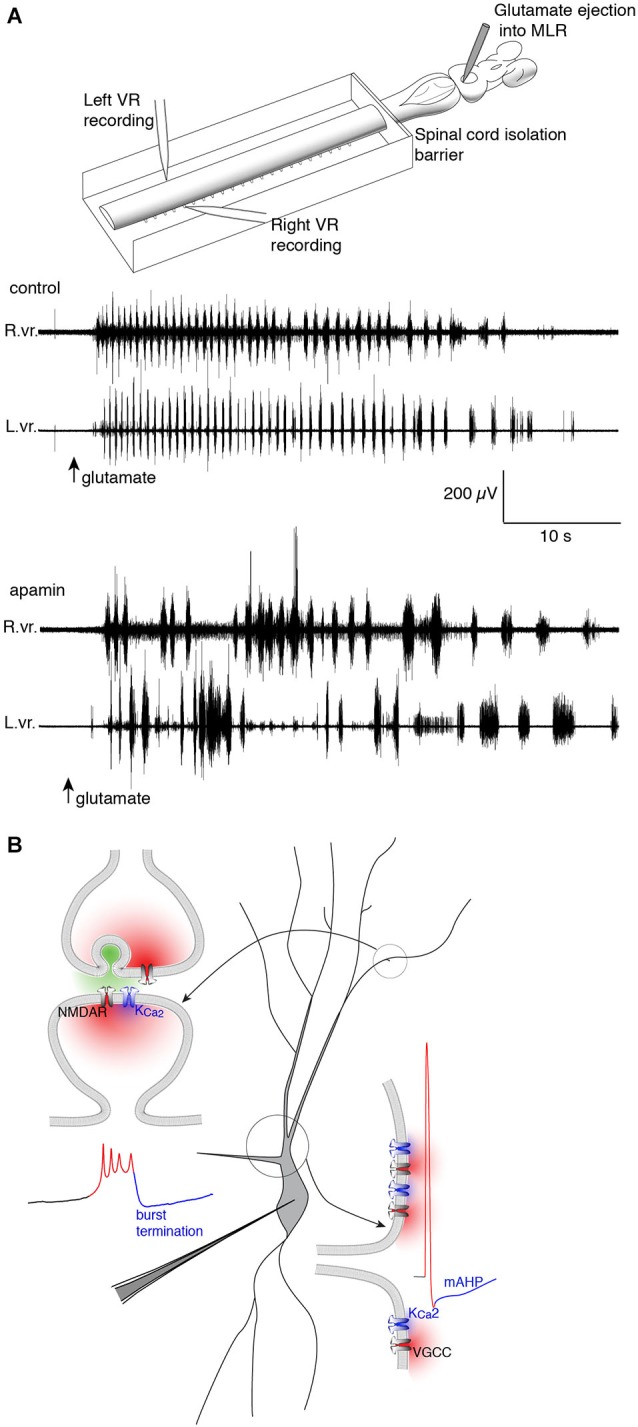
**Repolarizing K_Ca_2 channels are spatially segregated in lamprey spinal VHNs according to their function and mechanism of activation. (A)** Top: An isolated lamprey CNS can be used to study the brain and spinal circuits controlling locomotion. Pressure-ejection of L-glutamate into the lamprey mesencephalic locomotor region (MLR) induces short episodes of fictive locomotion, the electrophysiological correlate of locomotion. Using a dual-pool recording chamber, pharmacological agents can be selectively applied to the spinal cord, without interfering with descending commands originating in the brainstem that initiate and maintain locomotion. Locomotor bursts are recorded directly from left and right VRs. Bottom: A long locomotor episode with regular, alternating bursts (control) follows after a puff of glutamate into the MLR (arrow, glutamate). Blockade of K_Ca_2 channels with the selective antagonist, apamin, decreases the burst frequency and disrupts the alternating locomotor rhythm. This demonstrates the necessity of K_Ca_2 channels for correct alternation and regularity of the locomotor rhythm (Nanou et al., 2013). **(B)** The effect of K_Ca_2 channel blockade on locomotion can be explained by the role the channel plays at the cellular level. Within VHNs, K_Ca_2 currents may be evoked either at synapses (top left) whereby synaptic release of glutamate activates NMDAR-mediated Ca^2+^ entry and thereby closely located K_Ca_2 channels. It is this K_Ca_2-mediated current that is critical for the termination of NMDA-TTX oscillations (blue portion of trace) shown below recorded from somatic microelectrode recordings. K_Ca_2-mediated currents are also responsible for the mAHP seen following action potential firing shown at bottom left. However, this current is activated following Ca^2+^ entry from VGCCs.

The Ca^2+^-dependent K^+^ channel of the K_Ca_2 subtype (formerly SK2 (Wei et al., 2005)) participates in two distinct processes in lamprey VHNs both of which are integral to the behavioral locomotor output of the spinal cord. Its most well-described role follows the AP when depolarization activates N- and P/Q-type (Wikström and El Manira, 1998) voltage-gated Ca^2+^ channels (VGCCs) and the entering Ca^2+^ activates K_Ca_2 channels to cause an afterhyperpolarization (AHP; Figure 3; Hill et al., 1992; Meer and Buchanan, 1992). The AHP can be divided into fast, medium and slow subcomponents, of which the medium AHP (mAHP) is mediated by K_Ca_2 channels (Bond et al., 2004). Due to slow kinetics (decay time constant of ~200 ms), the mAHP mediates spike frequency adaptation, the reduction in spike frequency from repeated spiking, by raising the relative threshold for subsequent AP generation due to an increase in K^+^ conductance. Blockade of K_Ca_2 channels with the selective antagonist, apamin, increases spike frequency from intracellular current pulses (Meer and Buchanan, 1992; Díaz-Ríos et al., 2007; Jones and Stuart, 2013). K_Ca_2 channels are extremely important for regulating neuronal firing, conserved among different species and cell types (Meer and Buchanan, 1992; Sah and Bekkers, 1996; Marrion and Tavalin, 1998; Wikström and El Manira, 1998; Faber and Sah, 2002; Bloodgood and Sabatini, 2007; Jones and Stuart, 2013).

The second role for K_Ca_2 lies in the plateau termination and membrane repolarization during NMDA-TTX oscillations (Figure 3). The ionic mechanism driving *V_m_* oscillations is well-characterized and is hypothesized to proceed as: (1) NMDAR activation depolarizes VHNs; (2) increasing NMDAR conductance by ejecting Mg^2+^ from the pore; (3) causing further depolarization and Ca^2+^ entry via the NMDAR as the *V_m_* plateaus; (4) which activates K_Ca_2 channels to hyperpolarize the cell; and (5) ending the depolarized plateau to repolarize the cell where it can repeat the cycle (Wallén and Grillner, 1987). Selective blockade of K_Ca_2 channels with apamin (El Manira et al., 1994) or UCL 1684 (Alpert and Alford, 2013) prolongs the oscillation, and can even abolish the oscillation completely. The cell becomes trapped in a depolarized state, similar to extracellular Ca^2+^ removal, the substitution of Ca^2+^ for Ba^2+^, or non-specific blockade of K^+^ channels (Grillner and Wallén, 1985; Grillner et al., 2001). Thus, K_Ca_2 channels are necessary for rhythmogenesis (Figure 3) in lamprey VHNs by supplying a cell-autonomous repolarization, or “off signal”, without the need of network inhibition (Nanou et al., 2013).

## Dendritic Ca^**2+**^ signaling is dynamic and determined by cellular and microcircuit properties

K_Ca_2 channels within a single neuron have more than one distinct computational role. Two have been identified in lamprey VHNs, both subject to intracellular Ca^2+^ dynamics. Such a functional sub-specialization may be explained both by distinct spatial locations of channel expression and the adequate spatial and functional coupling to distinct sources of Ca^2+^ contributing to K_Ca_2 activation (Figure 3). Indeed, N- and P/Q-type (Wikström and El Manira, 1998) VGCCs are activated during the AP in lamprey, triggering Ca^2+^ entry that activates K_Ca_2 channels underlying the mAHP. However, the mAHP activated by somatic current injection is unaffected by NMDA application (Hill et al., 1989). This distinct separation between mAHP activation and NMDA-TTX oscillation repolarization can be explained by NMDAR-generated Ca^2+^ entry occurring in spatially distinct cellular sub-regions from VGCC-generated Ca^2+^ entry during the AP. Across different species and neuron types, the precise subtypes of VGCCs can differ, but to mediate the mAHP, K_Ca_2 channels must be sufficiently close to VGCCs to be activated by their Ca^2+^ permeation. Similarly, K_Ca_2 channels mediating repolarization during NMDA-TTX oscillations should be coupled to a distinct Ca^2+^ source, or a Ca^2+^ source in a distinct subcellular location. The two likely candidates for the latter are NMDARs and VGCCs (Wallén and Grillner, 1987)—located separately from those responsible for the mAHP (Hill et al., 1989)—while Ca^2+^ released from internal stores might also contribute. NMDAR activation is necessary to initiate oscillations, but as they lead to membrane depolarization, this may subsequently activate VGCCs. However, release from internal stores likely contributes little because their depletion has no effect on NMDA-induced swimming (Krieger et al., 2000)—a behavior to which NMDAR-dependent intrinsic oscillations contribute. The subcellular location of K_Ca_2 channels responsible for the repolarization may also be critical because physiological NMDAR activation requires the presynaptic release of glutamate, which occurs only at synapses. Determining the route of Ca^2+^ entry for repolarization of the oscillation is important for understanding how distinct Ca^2+^ domains and their coupling to K_Ca_2 channels impacts computation both within individual neurons and between synaptically connected neurons.

The spatial and temporal patterning over which dendritic Ca^2+^ signaling occurs in spinal motor system VHNs during locomotion in lamprey (or in other vertebrate systems) is unknown. Do many dendrites receive synchronous input from their various synaptic partners? Does input occur in discrete spatial locations? The location and timing of synaptic input is crucial for the transmission of potentials arriving at the soma, which will greatly influence neuronal output (Larkum et al., 1999; Stuart and Häusser, 2001; Jarsky et al., 2005). Indeed, dendritic mechanisms that are location-dependent and rely on clustered NMDAR-dependent input generate plateau potentials and can change the mode of cell firing (Major et al., 2008; Augustinaite et al., 2014; Grienberger et al., 2014). Elucidating this pattern within lamprey spinal neurons will inform how the location and timing of Ca^2+^ entry leads to K_Ca_2 channel activation, and furthermore, how synaptic activity distributed across a dendritic tree is integrated to produce cell rhythmicity. This, in turn, will facilitate our understanding of how intrinsic membrane properties combined with synaptic input causes synchronization between neurons of the CPG. Neuronal Ca^2+^ signaling can have distinct spatial components, easily identifiable using Ca^2+^ imaging. APs will lead to Ca^2+^ entry wherever VGCCs are driven above threshold, and can cause many regions of a cell (e.g., soma and proximal dendrites) to show synchronized increases in intracellular Ca^2+^ (Ca^2+^_i_). In contrast, local Ca^2+^ signaling domains (i.e., micro- and nano-domains) in dendrites can occur following neurotransmitter receptor activation (e.g., NMDAR), but also from VGCCs following depolarization from local synaptic potentials (Augustine et al., 2003). Synaptic signals are remarkably localized, confined to individual dendritic spines or discrete areas in dendritic shafts. For this reason, Ca^2+^ imaging can directly identify active synapses. Each type of Ca^2+^ signaling domain may be considered to be a distinct processing unit within a neuron because Ca^2+^ signals can regulate local Ca^2+^-dependent processes precisely where free Ca^2+^ levels transiently escape local buffering. However, Ca^2+^ signals exceeding this local threshold are transient—Ca^2+^ is rapidly buffered by Ca^2+^-binding proteins, and then extruded via membrane pumps, or sequestered in intracellular stores (Augustine et al., 2003; Berridge, 2006). This places temporal and spatial restrictions on diffusion of Ca^2+^ within neurons and is an important consideration when assessing the degree of localization. Dendritic morphology, like the presence of spines (~1 μm in length), is a large determinant for the extent of spread of Ca^2+^ because diffusion is restricted at the spine neck (Nimchinsky et al., 2002). Lamprey spinal neuron dendrites lack spines, but still posses fine compartments along dendritic shafts (~10 μm, see Figure 1; Viana di Prisco and Alford, 2004; Alpert and Alford, 2013) that may theoretically serve a similar purpose—the local restriction of the flow of ions and intracellular messengers (Svoboda et al., 1996). Thus, morphology and the intrinsic properties of the dendritic membrane impacts Ca^2+^ dynamics and the integration of electrical and chemical signals.

The functional distinction between global and local Ca^2+^ signals and their associated topography is integral to single neuron computation necessary to generate rhythmic activity. The synaptic localization of Ca^2+^ signals may represent the encoding of distinct presynaptic information. Global, synchronized Ca^2+^ signals can be generated by back-propagating action potential (bAP)-driven VGCC activation in dendrites (Schiller et al., 1997; Stuart et al., 1997; Svoboda et al., 1997). When Ca^2+^_i_ is elevated during these events, the number of parallel computations being performed by the dendritic arbor is effectively reduced. In contrast, local and spatially distributed NMDAR-dependent synaptic Ca^2+^ signals reflect multiple discrete, simultaneous computations (Chen et al., 2011). Each synapse can thus be understood to be its own computational unit, capable of being selectively tuned to support distinct information arriving within a network.

Multiple, distinct routes can lead to Ca^2+^ entry. In behaving neurons within some networks, these mechanisms may work in concert, leading to nonlinear interactions between ion channels and Ca^2+^ sources when occurring simultaneously. For instance, following presynaptic release of glutamate, AMPA receptors (AMPARs), NMDARs and metabotropic glutamate receptors (mGluRs) may be activated in the postsynaptic compartment. AMPARs are responsible for fast depolarization, and can locally activate nearby VGCCs to cause Ca^2+^ entry. Local depolarization, or depolarization induced from bAPs can alleviate Mg^2+^ block of the NMDAR, facilitating Ca^2+^ influx during concurrent and subsequent release of glutamate at that synapse (Yuste and Denk, 1995; Nevian and Sakmann, 2004; Bloodgood and Sabatini, 2007). During bAPs, layer 5 pyramidal neurons have been shown to require tight spatial coupling between Ca^2+^ entry through R-type channels and K_Ca_2 channels in proximal dendrites and spines (Jones and Stuart, 2013). Group I mGluR activation can lead to the release of Ca^2+^ from internal stores (Frenguelli et al., 1993; Kettunen et al., 2002; Larkum et al., 2003; Topolnik et al., 2009; Plotkin et al., 2013). Release from internal stores has been shown to activate Ca^2+^-dependent K^+^ channels in many neurons and species (Kawai and Watanabe, 1989; Akita and Kuba, 2000; Yamada et al., 2004; Faber, 2010; Nakamura and Yokotani, 2010). It is unknown if such combinatory mechanisms are present in lamprey spinal neurons, but lamprey neurons do possess all the necessary components. Indeed, specific agonists and antagonists acting on discrete components have well-described cellular and network effects (Alford et al., 2003). Any modulation of Ca^2+^ entry, either increasing or decreasing, within close proximity to K_Ca_2 channels, could impact subsequent channel activation and particular effects on the locomotor behavior. For example, an enhancement of Ca^2+^ could lead to early burst termination—an effect that, if it were to occur within many neurons simultaneously, would scale to the behavioral level to terminate muscle contraction earlier within the locomotor cycle. Upon repeated enhancements in Ca^2+^, during rhythmic activity, this could facilitate a fast swimming rhythm. Defining their roles while acting in concert is necessary to delineate how Ca^2+^ entry and K_Ca_2 activations drives oscillation generation.

The location of Ca^2+^ entry and the distance to its secondary effectors determines the efficacy with which Ca^2+^ will reach its target (Marrion and Tavalin, 1998). If the site of Ca^2+^ entry is located far from K_Ca_2 channels, then the probability of Ca^2+^ binding to a K_Ca_2 channel is diminished compared to its binding to other endogenous buffers that are located more proximally or are cytosolic and diffusible. Thus, a larger Ca^2+^ signal will be necessary to outcompete endogenous buffers. Conversely, if K_Ca_2 channels are located close to the site of Ca^2+^ entry, then depolarization will be quickly and locally counteracted by K^+^ activation. For K_Ca_2 channels to generate the mAHP, they must be sufficiently close to the site of Ca^2+^ entry generated by AP-driven VGCC activation. This functional coupling has been demonstrated in numerous species and cell types (Sah and Bekkers, 1996; Marrion and Tavalin, 1998; Wikström and El Manira, 1998; Faber and Sah, 2002; Bloodgood and Sabatini, 2007; Jones and Stuart, 2013). At present, the distance between the site of Ca^2+^ entry and K_Ca_2 channels can only be estimated based on differences between BAPTA and EGTA-mediated occlusion of K_Ca_2 activation in lamprey spinal VHNs. The range has been estimated to be between 20 and >200 nm in multiple CNS neuron types depending on the target’s affinity for Ca^2+^ (Fakler and Adelman, 2008). A recent measurement has suggested that K_Ca_2 channels activated following APs exhibit weak coupling to VGCCs, as they are occluded by EGTA, the slow Ca^2+^ buffer (*K_forward_* = 1.5 × 10^6^ M^−1^s^−1^) (Roberts, 1993), placing the separation at greater than ~100 nm in neocortical pyramidal neurons (Jones and Stuart, 2013). Occlusion of K_Ca_2 channel activation from NMDAR-dependent Ca^2+^ entry using BAPTA, the fast Ca^2+^ buffer (*K_forward_* = 6 × 10^8^ M^−1^s^−1^) (Roberts, 1993), demonstrates a narrow range of 20–50 nm (Ngo-Anh et al., 2005), with experiments in lamprey suggesting similar degree of coupling (Alpert and Alford, 2013; Nanou et al., 2013).

For K_Ca_2 channels to repolarize NMDA-TTX *V_m_* oscillations, they must be activated by NMDAR-dependent Ca^2+^ entry. The subcellular expression of ion channels, including K_Ca_2 channels, is unknown in lamprey, while some spatial information has been detailed for mammalian hippocampal neurons. K_Ca_2 channel immunoreactivity demonstrates channel expression on dendritic spines in CA1 pyramidal neurons (Sailer et al., 2004; Ballesteros-Merino et al., 2012) in addition to shafts and soma in cultured mice hippocampal neurons (Ngo-Anh et al., 2005). Recently, however, using single-molecule atomic force microscopy with unprecedented spatial resolution (<10 nm (Müller et al., 2009)), K_Ca_2 channels were shown to be in high concentration in the dendrites relative to the soma of live, cultured hippocampal neurons (Maciaszek et al., 2012). Functional evidence for complexes of NMDARs and K_Ca_2 channels has been demonstrated in many species and cell types. NMDAR-mediated field potentials are potentiated by apamin in CA1 hippocampal pyramidal neurons (Gu et al., 2008). Direct NMDA application leads to an inward current followed by an apamin-dependent outward current (Shah and Haylett, 2002; Nanou et al., 2013). Apamin potentiates both synaptically evoked NMDAR EPSPs on CA1 dendrites, while also potentiating apical spine Ca^2+^ transients (Ngo-Anh et al., 2005). However, it was later demonstrated using 2-photon glutamate uncaging that Ca^2+^ entry via R-type VGCCs is necessary and directly coupled to K_Ca_2 channels at these spine synapses, whereas NMDAR-dependent Ca^2+^ is insufficient to activate K_Ca_2 channels (Bloodgood and Sabatini, 2007). This discrepancy was recently reconciled with experiments demonstrating that K_v_4.2-containing channels and NMDARs are differentially coupled to R-type VGCCs and NMDARs, respectively (Wang et al., 2014b). Furthermore, K_Ca_2 channel activation by NMDAR-induced spine Ca^2+^ transients is also occluded by BAPTA, but not by EGTA, indicating a very close physical coupling of the route of Ca^2+^ entry and the K_Ca_2 channel (Ngo-Anh et al., 2005)—a similar role for NMDARs and K_Ca_2 channel is also demonstrated in the lateral amygdala (Faber et al., 2005) and in layer 5 neocortical pyramidal neurons (Faber, 2010). Furthermore, overexpression of K_Ca_2 channels depresses synaptically evoked glutamatergic EPSPs (Hammond et al., 2006). Due to the role of NMDAR-dependent Ca^2+^ entry in synaptic plasticity, blockade of K_Ca_2 channels facilitates the induction of LTP (Stackman et al., 2002) because this, in turn, facilitates Ca^2+^ entry through NMDARs, presumably by augmenting depolarization. Similarly, downregulation of K_Ca_2 channels is necessary for amplification of dendritic responses in a compartment- (Ohtsuki et al., 2012) or synapse-specific (Lin et al., 2008) manner, partially explaining the subsequent potentiation of current.

Thus, the very precise subcellular targeting of K_Ca_2 channels to ion channels responsible for Ca^2+^ transients (demonstrated by sensitivity to rapid Ca^2+^ binding by BAPTA) will profoundly impact cell firing rates, dendritic integration, and processing both in real-time during individual cycles of locomotor activity, but also in the long-term. The molecular complexing of Ca^2+^ sources to secondary effector proteins, like K_Ca_2 in lamprey, will consequently impact spike-timing through activation of the mAHP (Buchanan and Grillner, 1987; Wallén and Grillner, 1987; Alford and Williams, 1989; Wallén et al., 1989; Hill et al., 1992; Hochman et al., 1994; Wall and Dale, 1995; Buchanan, 2001; Harris-Warrick, 2002; Wang, 2010) in addition to burst termination (El Manira et al., 1994; Alpert and Alford, 2013; Nanou et al., 2013) during NMDAR-dependent rhythmic activity. These intrinsic membrane properties have direct consequences on spinal neuron output, and hence the locomotor pattern generation of the spinal network.

## Evidence for a dendritic mechanism of intrinsic oscillations in the CNS

In lamprey VHNs filled with a Ca^2+^-sensitive dye, Ca^2+^_i_ oscillates in-phase with VR bursts and *V_m_* oscillations, varying with different NMDA-induced swimming speeds (Bacskai et al., 1995; Viana di Prisco and Alford, 2004). In contrast, during activity that was subthreshold to action potential firing, the soma showed no Ca^2+^ fluctuations, while the dendritic fluorescence oscillated in-phase with the *V_m_*, with the largest oscillations in Ca^2+^_i_ found in the distal dendrites. When spiking, the somatic Ca^2+^ then showed spike-dependent activity, which is in-phase with *V_m_*, oscillations because spiking occurs at the depolarized phase of activity. However, despite spikes also elevating dendritic Ca^2+^ signals, the largest increases in fluorescence occurred in the soma, likely reflecting somatically localized VGCC activation (Viana di Prisco and Alford, 2004). The elevated dendritic signals could be due to dendritic VGCC activation, enhanced NMDAR conductance due to local depolarization from bAPs, or both. The phase relationships of the Ca^2+^ oscillations in dendrites relative to *V_m_* oscillations suggest that these Ca^2+^ signals are responsible for K_Ca_2 activation, and hence repolarization of the membrane. This result along with experiments discussed earlier (Grillner and Wallén, 1985; Wallén and Grillner, 1987) provide substantial evidence that NMDAR-dependent Ca^2+^ entry underlies the repolarization of *V_m_* oscillations.

**Figure 4:**
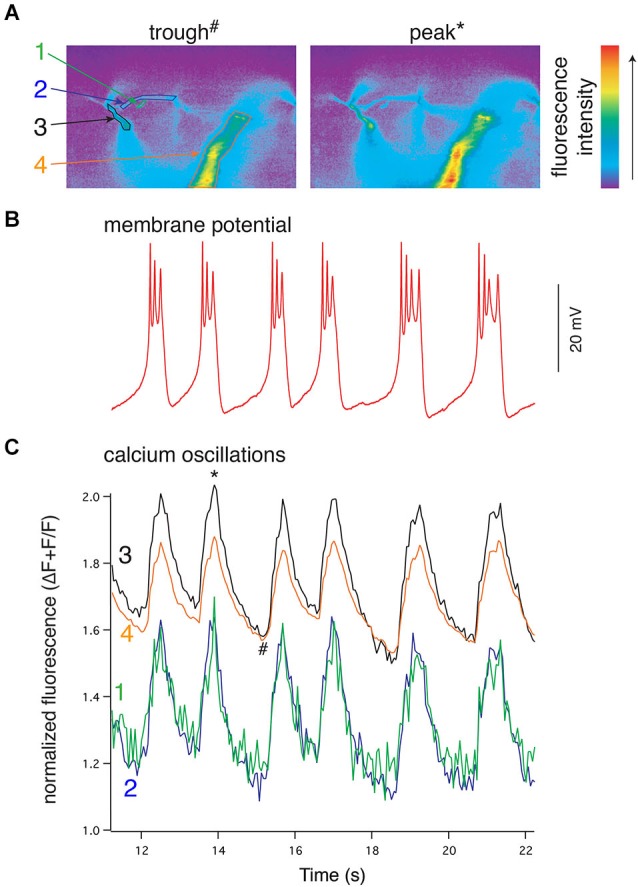
**NMDA-evoked, TTX-resistant oscillations in lamprey VHNs show simultaneous oscillations in Ca^2+^ throughout the dendritic tree. (A)** VHN neurons were labeled with the Ca^2+^-sensitive dye, Oregon Green 488 BAPTA1, by pressure injection from a recording microelectrode and recorded during oscillations evoked by application of NMDA (100 μM) in TTX (1 μM). Pseudocolored, raw images are shown from the trough of the hyperpolarization (left, denoted by # in **(C)**) and the peak of depolarization (right, denoted by * in **(C)**). Colored numbers and arrows point to discrete regions of interest whose fluorescence measurements are shown in **(C)**. Fluorescence intensity scale shown to the right. **(B)** Current clamp recording of the membrane potential oscillations. **(C)** Simultaneous to the membrane potential oscillations in **(B)**, Ca^2+^ recorded using the fluorescent dye Oregon Green 488 BAPTA1 shows transient increases in concentration in the dendrites. In the proximal dendrites, the oscillations are above a higher baseline Ca^2+^ evoked by NMDA application than that recorded in the distal dendrites, however, all recorded regions of the dendrites exhibit these Ca^2+^ oscillations. All Ca^2+^ fluorescence is normalized to the fluorescence at rest prior to the application of NMDA. The regions recorded are indicated by colored numbers in **(A)** and **(C)** (Alford et al., 2003).

Results from experiments in which the spinal CPG is activated by application of exogenous NMDA also imply that rhythmic *V_m_* oscillations are driven by phasic Ca^2+^ oscillations that are synchronized across large regions, if not all, of the dendritic tree (Figure 4). However, during bath-application of NMDA, both synaptic and extrasynaptic NMDARs are activated and thus the dendritic Ca^2+^ signals are likely to be much less spatially and temporally constrained than signals driven during physiologically evoked locomotion. This forces the concerted activation of all NMDARs when the dendritic membrane is depolarized, which would consequently synchronize all parts of the neuron. Thus, it is unclear if during NMDA-evoked locomotion whether network synchrony is driven by synchronized presynaptic activity caused directly by bath-applied NMDA, or if rhythmicity emerges from more physiologically derived synaptic integration of distributed input and is then transformed into well-defined *V_m_* oscillations. Similarly, the spatiotemporal profile of dendritic activation and Ca^2+^ signaling underlying membrane potential oscillations during locomotion remains unknown. This profile will, however, be particularly important for understanding how membrane properties drive the activity of the network.

Synchronized oscillations are widespread in the CNS. While critical for the generation of motor rhythms, they are key components of many neural systems. In the neocortex and hippocampus, oscillations at the cellular level are correlated with synchrony at the network level (Contreras and Steriade, 1995) and are thought to underlie cognitive processes such as working memory (Llinás, 1988), spatial navigation, and memory encoding (Buzsáki and Moser, 2013). Both theoretical approaches and experimental evidence suggest that the cellular basis for working memory relies upon persistent firing of networks generated by recurrent synaptic excitation of NMDARs due to its slow kinetics and voltage-dependency (Lisman et al., 1998; Wang, 2001) conferring bistability (Durstewitz et al., 2000). NMDA-TTX oscillations are also found in midbrain dopamine neurons (Johnson et al., 1992; Deister et al., 2009) cat neocortical pyramidal neurons (Flatman et al., 1986), rat inferior olivary neurons (Placantonakis and Welsh, 2001), *Xenopus* RS neurons (Li et al., 2010), guineau pig and rat trigeminal motor neurons (Kim and Chandler, 1995; Hsiao et al., 2002), and rat and cat thalamocortical neurons (Leresche et al., 1991), demonstrating a similar intrinsic oscillatory mechanism to lamprey spinal neurons.

Although arrangements involving NMDARs and K_Ca_2 channels have been shown in many other systems and synapses, their functions have not been expressly linked to specific behaviors or to rhythm generation, but rather have been proposed to serve a more generalized mechanism for tempering synaptic potentials and synaptic plasticity (Shah and Haylett, 2002; Stackman et al., 2002; Maher and Westbrook, 2005; Ngo-Anh et al., 2005; Gu et al., 2008; Lin et al., 2008; Faber, 2010; Harvey-Girard and Maler, 2013). Apamin or intracellular dialysis with BAPTA prolongs glutamate-induced plateau potentials and Ca^2+^ transients in CA1 pyramidal neuron distal apical dendrites (Wei et al., 2001; Cai et al., 2004), but these plateau potentials are produced through a combination of R-type VGCCs, NMDARs and bAPs (Takahashi and Magee, 2009), and thus does not explicitly link Ca^2+^ entry via NMDARs to K_Ca_2 activation. NMDAR-dependent activation of K_Ca_2 channels at layer 5 pyramidal neuron synapses (Faber, 2010) indicates the necessary components are present, but the mechanism of dendritic oscillation generation has yet to be explored. Recently, a dendritic mechanism for synchronizing spatially disparate synaptic input has been described in CA1 pyramidal neurons, which has implications in the synchronization of hippocampal networks (Vaidya and Johnston, 2013). In lamprey spinal neurons, the nonlinear dynamics of the NMDAR combined with its spatial coupling to K_Ca_2 channels confers *V_m_* bistability, enabling these neurons to be oscillators to synchronize spinal networks for rhythmic locomotion.

## Evidence for close coupling of NMDARs and K_Ca_2 channels

If NMDARs are the primary route of Ca^2+^ entry necessary for repolarization, then synaptically activated NMDARs will evoke highly localized Ca^2+^ entry within spinal neuron dendrites, and this Ca^2+^ must be located sufficiently close to K_Ca_2 channels to activate an outward current. EIN stimulation causes localized, NMDAR-dependent Ca^2+^ entry in VHN dendrites (Alpert and Alford, 2013). NMDAR EPSCs are sufficient to activate K^+^ currents, which are blocked by the K_Ca_2 blockers, apamin and UCL 1684, or following whole cell dialysis with BAPTA. In contrast, EGTA dialysis is ineffective at preventing K_Ca_2 activation (Nanou et al., 2013). Furthermore, BAPTA also prevents repolarization from depolarized plateaus in oscillating neurons induced by NMDA in TTX, whereas those dialyzed with EGTA are able to repeatedly repolarize (Alpert and Alford, 2013). Since BAPTA, but not EGTA, occludes the binding of Ca^2+^ to secondary effectors, presumably K_Ca_2, the channel must be located physically close to the site of Ca^2+^ entry (Marrion and Tavalin, 1998; Ngo-Anh et al., 2005; Fakler and Adelman, 2008).

Any possible role for VGCCs in directly providing Ca^2+^ to drive the repolarization is somewhat limited by the voltage threshold of activation relative to the *V_m_* oscillation range. Lamprey VHNs contain multiple subtypes of VGCCs including N-, P/Q-, and L-type channels with varying contributions to depolarization-evoked whole-cell currents1 (El Manira and Bussières, 1997) and presumably distinct cellular localizations (Llinás and Yarom, 1981; Llinás, 1988; Westenbroek et al., 1990, 1992; Mills et al., 1994; Isope et al., 2012). In cultured lamprey spinal neurons, N- and P/Q-type channels account for ~75% of the total whole cell VGCC current, while L-type current contributes ~15% with the residual Ca^2+^ current uncharacterized, but sensitive to Cd^2+^, the non-specific VGCC blocker (El Manira and Bussières, 1997). However, these values are likely impacted by reduced dendritic arbors in culture and space clamp issues common to somatic recordings. Cd^2+^ abolishes whole-cell current *in situ*, yet NMDA-TTX oscillations persist in Cd^2+^ (Alpert and Alford, 2013), while simultaneous Ca^2+^_i_ oscillations are insensitive to selective blockade of N- and P/Q-type VGCCs. L-type channels couple to K_Ca_2 channels in hippocampal pyramidal neurons (Marrion and Tavalin, 1998), while Ca^2+^ imaging suggests that this coupling may exist in a subset of dendritic loci in lamprey (Wang et al., 2013) because modulation of L-type channels impacts Ca^2+^ oscillations and the *V_m_* oscillation waveform (Wang et al., 2014a). The current-voltage (I-V) relationship of VGCCs in VHNs shows minimal activation at −60 mV, with significant activation occurring between −40 mV and −30 mV, peaking between −10 and 0 mV (El Manira and Bussières, 1997; Alpert and Alford, 2013). Interestingly, the same *V_m_* where VGCCs become activated, −40 mV, is also the peak plateau potential reached during membrane potential oscillations in NMDA (Alpert and Alford, 2013). Thus, for the majority of VHNs, the neurons oscillate subthreshold to the VGCC activation thresholds except for the initial transient peak of the oscillation amplitude. Indeed, NMDA application reveals a depolarizing step-evoked inward current that occurs within the *V_m_* oscillation range at substantially more hyperpolarized *V_m_*s than currents mediated by VGCCs in these neurons. Similarly, Ca^2+^-imaging indicates that Ca^2+^ entry within the oscillation range is robustly potentiated and dominated by NMDAR-dependent Ca^2+^ entry (Alpert and Alford, 2013). During voltage steps in NMDA, biphasic currents are generated. This NMDA-induced inward current followed by an outward current is present within the oscillation range (i.e., below −40 mV) and blocked by BAPTA, but not EGTA, again reflecting a close functional coupling between NMDAR current and presumably K_Ca_2 channel activation leading to the outward current. Therefore, both *V_m_* and Ca^2+^_i_ oscillations are driven through a dendritic mechanism requiring closely apposed ensembles of NMDARs and K_Ca_2 channels and little contribution of Ca^2+^ from VGCCs.

## Dendritic structure and synaptic integration of presynaptic microcircuitry of ventral horn neurons

In general, the origin of presynaptic input, synapse location within the dendritic tree, and electrotonic distance to soma informs the computation performed by the postsynaptic neuron. Spatially and anatomically compartmentalized dendritic targeting by presynaptic axons is found in many vertebrate neural circuits including the tectum (Bollmann and Engert, 2009), hippocampus (Pouille and Scanziani, 2004; Jarsky et al., 2005), neocortex (Weiler et al., 2008; Anderson et al., 2010), and cerebellum (Ito, 2006; Gao et al., 2012). Variability in presynaptic activity can lead to variation of the topology of Ca^2+^ signaling postsynaptically where it may be encoded predictably onto distinct dendritic compartments (Bollmann and Engert, 2009; Xu et al., 2012). In other instances, Ca^2+^ signaling is unpredictably encoded and may demonstrate extremely heterogeneous expression of activity, even at neighboring synapses (Chen et al., 2011). Global Ca^2+^ signals generated by AP-induced VGCC activation may appear qualitatively similar to those generated from convergent presynaptic activation leading to a global rise in Ca^2+^. However, the computation performed by a neuron is distinct, depending on the modality of Ca^2+^ signaling. Somatic signals provide intrinsic information about cell firing, while local, synaptic signals inform about the spatial and functional connectivity of the network and its activation state. Indeed, the computational ability of a neuron’s dendrites is intimately tied to and ultimately informed by presynaptic inputs, whose activity leads to discernable behavioral functions postsynaptically (Jia et al., 2010). In hippocampal and neocortical pyramidal neurons, neighboring dendritic synapses are more likely to be activated synchronously than synapses spaced further apart (Kleindienst et al., 2011; Takahashi et al., 2012). This functional clustering is NMDAR-dependent and likely due to synchronized and convergent targeting of multiple presynaptic axons projecting to the recorded neurons, rather than a single presynaptic axon making multiple contacts. Such functional clustering may be important for circuit orchestration during development (Kleindienst et al., 2011) and experience-dependent synaptic plasticity (Makino and Malinow, 2011). Furthermore, NMDAR activation is essential for nonlinear boosting of temporally and spatially integrated synaptic potentials (Polsky et al., 2004). Synaptic potentials arriving at the soma from discrete synaptic events can vary according to degree of clustering (Losonczy and Magee, 2006) and the direction and velocity of synaptic input along single dendritic branches (Branco et al., 2010). The patterning of synaptic input has profound consequences on Ca^2+^_i_ and this “within dendritic branch” form of computation is NMDAR-dependent. Furthermore, synaptic plasticity—the Ca^2+^-dependent change in strength of a synapse—can occur selectively at a single synapse (Matsuzaki et al., 2004; Enoki et al., 2009; Makino and Malinow, 2011). Thus, postsynaptic responses to Ca^2+^, and hence dendritic computational capacity (Poirazi and Mel, 2001), are highly dynamic and depend on presynaptic input and subsequent post-synaptic Ca^2+^ signals as well as the function of the circuit in which the neuron is embedded.

Discrete targeting provides neurons with more processing power (Häusser and Mel, 2003; Polsky et al., 2004) by integrating origin-specific, segregated streams of presynaptic information. This is further enhanced as the location and expression of various voltage-gated ion channels and synaptic receptors varies between different types of neurons but also subcellularly, between different regions of a single neuron (Migliore and Shepherd, 2002; Williams and Stuart, 2003; Jones et al., 2014). Such circuit and dendrite dynamics may also be present in spinal networks controlling movement. A well-defined topographic map of spinal motoneuron recruitment in larval zebrafish proceeds from the ventral to dorsal spinal cord as swimming frequency increases (McLean et al., 2007) and neurons are recruited functionally according to intrinsic rhythm-generating capabilities and requirement for presynaptic oscillatory synaptic drive (Menelaou and McLean, 2012). However, the interneurons that drive motoneuron recruitment demonstrate more complex activation patterns (McLean et al., 2008). The spatial targeting of motoneuron or interneuron dendrites and the integration of synaptic inputs conferring rhythmicity have yet to be defined, but dendritic filopodial activity follows a topographic pattern that maps (Kishore and Fetcho, 2013) onto their recruitment order (McLean et al., 2007) and subsequent electrical activity level, delineating behavioral function to dendrites located in discrete regions along the dorso-ventral axis. Thus, the location and targeting of specific dendritic subregions by spatially defined presynaptic neurons may suggest a functional role for individual dendritic branches (Wei et al., 2001; Poirazi et al., 2003; Branco and Häusser, 2010), or perhaps even synapses (Jia et al., 2010), in the output of a given motor neuron.

Dendrite distribution has been shown to differ for motoneurons innervating distinct muscles in the chick spinal cord (Okado et al., 1990). Mice motoneuron dendrites are genetically oriented to particular spinal territories, which influence the connectivity patterns of their proprioceptive afferent inputs (Vrieseling and Arber, 2006). The targeting of dendrites into specific lamina provides distinct opportunities for different classes of presynaptic excitatory and inhibitory interneurons to also target different dendritic regions (Kosugi et al., 2013). *Drosophila* motoneuron dendrites are topographically organized whereby individual neurons genetically target their dendrites to precise anatomical territories centrally, representing their muscle distribution peripherally (Landgraf et al., 2003; Brierley et al., 2009). Within a single dendritic tree there can be a heterogeneous patterning of excitatory synapses (Mauss et al., 2009) and, furthermore, distinct dendritic subtrees can target discrete regions of the neuropil (Vonhoff and Duch, 2010). Therefore, organizational principles orchestrating spinal circuits controlling locomotion are subject to genetic, developmental, and activity-dependent specificity, but determining the function of distinct subcellular targeting requires further investigation.

## Synapse-specificity of K_Ca_2 channels is behaviorally relevant

**Figure 5:**
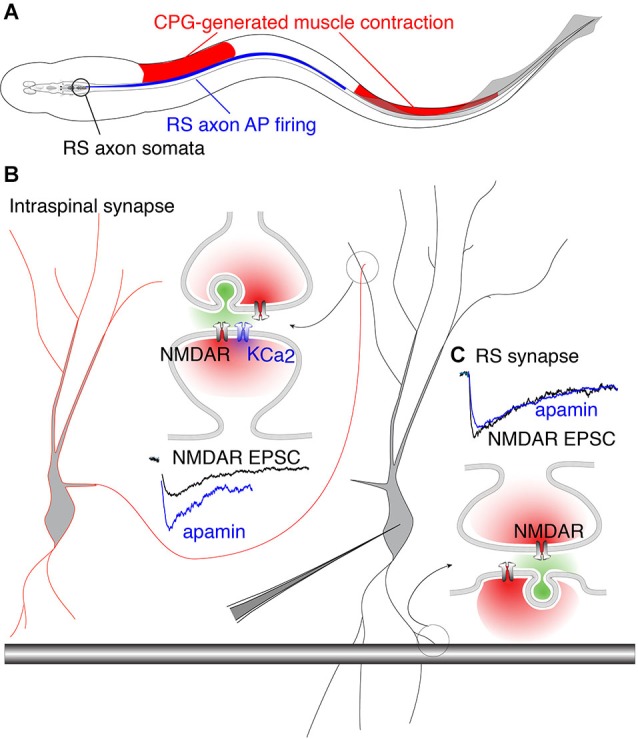
**Phase-matched and phase-mismatched excitatory synapses in the spinal cord of lamprey. (A)** As the lamprey swims it generates a traveling wave from head to tail. The sinusoidal body curvature illustrated here represents a single moment in body movement during a bout of swimming. During swimming, command excitation is continually provided by RS axons whose somata in the brainstem (encircled in black) fire (blue) in phase with the rostral spinal CPG neurons. This is illustrated by the rostro-caudal overlap of red and blue. It is the output of spinal segments that causes ipsilateral muscle contraction (red). Due to the speed at which the action potential (AP) propagates along RS axons, the AP invades more caudal areas of the spinal cord whose associated muscles do not undergo contraction because the CPG wave (responsible for contraction) travels at a delay relative to the AP. This leads to regions along the spinal cord where AP firing overlaps with inhibited musculature (illustrated by overlap of blue and white regions in the middle). This would predictably lead VHN excitation at inappropriate times during the swim cycle. This phase mismatch between RS axons and CPG neurons may be avoided by synapse-specific K_Ca_2 channel activation. **(B)** Circuit model in which excitation from EINs (red outlined cell) projects to other VHNs (black outlined cell) locally within the spinal cord. NMDAR currents from these neurons (black trace, NMDAR EPSC) are enhanced by the addition of apamin, the specific K_Ca_2 channel antagonist, to block K_Ca_2 currents (blue trace). **(C)** RS synapses from large descending axons (black shaded bar) which project throughout the spinal cord, show NMDAR currents (black trace, NMDAR EPSC) that are unaffected by apamin (blue trace) (Alpert and Alford, 2013).

The precise coupling of synaptically activated receptors and secondarily activated ion channels may complement anatomical specificity of excitatory connections. The behavioral importance of this coupling becomes evident when considering how descending brainstem RS neuron drive interacts with the spinal cord CPG. In vertebrates, RS neurons receive feedback modulation from the spinal CPG that causes them to fire in-phase with the rostral spinal segments (Kasicki et al., 1989; Sirota et al., 2000; Dubuc et al., 2008). This phenomenon creates a paradox with respect to RS innervation of the spinal CPG. In lamprey, each VHN receives input from both local circuit interneurons (glutamatergic and glycinergic) (Buchanan, 1982; Buchanan and Grillner, 1987) and descending RS axons (glutamatergic) (Buchanan et al., 1987; Brodin et al., 1988). Thus, a single VHN may receive two distinct types of glutamatergic contacts. Since the animal creates a rostro-caudal phase lag of 360° from head to tail (Wallén and Williams, 1984), substantial regions of the spinal cord are necessarily out-of-phase with RS neuron firing. Furthermore, as the fish swims, RS axon APs are initiated in the brainstem and project throughout the length of the spinal cord where they excite local CPGs. The AP propagation rate is faster than the speed of the mechanical wave driven by the propagation of neural excitation by segmental CPGs (~10 Hz traveling wave). Thus, there are two traveling waves, RS axon-generated AP propagation and CPG neural waves, which are out-of-phase across substantial rostro-caudal regions of the spinal cord and whose phase mismatch varies with locomotor frequency (Figure 5). This phase mismatch precludes RS axons from being phase-locked with VHNs, thus removing the need for pre- and postsynaptic synchronization conferred by coupling of NMDARs and K_Ca_2 channel activation.

Accordingly, it may be considered problematic for RS synapses expressing NMDARs to be coupled to K_Ca_2 channels, which would instill a strict phase-relationship between the pre- and post-synaptic neuron via excitation-inhibition coupling. In contrast, spinal EINs are appropriately phase-locked to their targets because the extent of their spinal projections are limited (Buchanan et al., 1989). This hypothesis is supported by experiments utilizing paired recordings between RS axons and VHNs, demonstrating that postsynaptic NMDAR-mediated responses are insensitive to apamin (Cangiano et al., 2002; Alpert and Alford, 2013). In contrast, glutamatergic synapses between EINs and other VHNs within the spinal cord exhibit strong NMDAR coupling to K_Ca_2 channels (Alpert and Alford, 2013; Nanou et al., 2013), conferring excitation-inhibition coupling and phase-locking as the CPG waves propagate between segments. Such synapse-specificity emphasizes the highly localized nature of dendritic Ca^2+^ signals and the profound importance for restricting Ca^2+^ entry within local domains (Figure 5). Within this framework, the synapse-specificity of K_Ca_2 channel activation is crucial for creating synchrony between neurons of the spinal network. Thus, the synaptic localization of the K_Ca_2 channel coupled to NMDARs is not just important for opposing depolarization, but together with the precise function of the presynaptic neuron, establish the foundation for generating network rhythmicity.

## Neuromodulation of K_Ca_2 channels mediating locomotion

Locomotion is also activated and modulated by monoaminergic systems. Bath-applied serotonin (5-HT), alone or within a cocktail of monoamines, can activate locomotion and fictive locomotion in many preparations (Cazalets et al., 1992; Rossignol et al., 2002). Like glutamate, spinal release of 5-HT originates from both intraspinal (Schotland et al., 1995, 1996; Zhang and Grillner, 2000) and brainstem neurons (Zhang et al., 1996; Abalo et al., 2007; Antri et al., 2008; Barreiro-Iglesias et al., 2008). In lampreys, bath-applied 5-HT has a well-defined modulatory effect on the CPG—it slows ventral root bursting during both spinal exogenous agonist-evoked (Wikström et al., 1995) and brainstem-evoked locomotion (Gerachshenko et al., 2009). 5-HT mediates its effects both pre- and post-synaptically through mechanistically distinct but behaviorally convergent effects. Postsynaptically, 5-HT modifies the activation of K_Ca_2 channels. This postsynaptic effect is mediated at two distinct subcellular sites. 5-HT suppresses burst termination during fictive locomotion induced by NMDA (Harris-Warrick and Cohen, 1985), an effect present during NMDA-TTX driven intrinsic oscillations (Wallén et al., 1989) and which is analogous to blockade of K_Ca_2 channels with apamin (El Manira et al., 1994) or UCL 1684 (Alpert and Alford, 2013). This prolongation of the oscillation may be mediated by direct interaction of 5-HT receptors on K_Ca_2 channels, or alternatively, via an indirect inhibition of NMDARs (Schotland and Grillner, 1993) or VGCCs (Wang et al., 2014a) supplying Ca^2+^ for K_Ca_2 channels responsible for the repolarization. Interestingly, the effects of 5-HT are absent when the network is activated by kainate, which will not activate NMDARs directly. This suggests that NMDAR-dependent Ca^2+^ entry contributing to burst termination (Alpert and Alford, 2013; Nanou et al., 2013) is necessary for 5-HT to modulate the oscillation and that 5-HT receptors inhibit K_Ca_2 channels activated directly by NMDAR-mediated Ca^2+^ permeation.

In addition to effects of 5-HT directly on NMDAR-mediated oscillations, 5-HT_1A_ receptors (Wikström et al., 1995) inhibit N-type VGCCs (Hill et al., 2003), reducing Ca^2+^ necessary for K_Ca_2 channel activation involved in mAHP (Wikström and El Manira, 1998). This effect is accordingly limited to individual neurons that spike repetitively during locomotion (or fictive locomotion). Thus, 5-HT interactions with K_Ca_2 channels are important in controlling firing rates in lamprey (Wallén et al., 1989; Hill et al., 1992; Meer and Buchanan, 1992) as well as other systems, but are also integral to the ionic mechanism contributing to NMDAR-dependent oscillatory properties (Harris-Warrick and Cohen, 1985; El Manira et al., 1994; Alpert and Alford, 2013; Nanou et al., 2013).

Presynaptically, 5-HT modulates glutamate release from intraspinal connections (e.g., EIN-VHN synapses) as well as from RS command neurons (Buchanan and Grillner, 1991; Blackmer et al., 2001, 2005). This effect mediated by 5-HT_1B_ receptors acts synergistically with the effects on K_Ca_2 channels. It also lengthens the locomotor burst duration during agonist- and (Schwartz et al., 2005) brainstem-evoked locomotion (Gerachshenko et al., 2009) by blocking synaptotagmin/SNARE complex interactions (Blackmer et al., 2005). This reduces cleft glutamate concentration, which leads to a selective reduction of AMPAR activation because NMDARs respond to low glutamate cleft concentrations more readily than do AMPARs (Patneau and Mayer, 1990; Choi et al., 2000; Schwartz et al., 2007). Sustained NMDAR activation combined with reduced AMPAR activation slows bursting recorded during fictive locomotion. This is similar to pharmacologically induced locomotion, which shows slower burst rates in NMDA compared to AMPA or kainate (Brodin et al., 1985; Alford and Grillner, 1990). It may in part be attributed to the slow and fast kinetics of NMDARs and AMPARs, respectively (Alford and Grillner, 1990), but is also a function of the spinal network in which repetitive activation that causes augmenting synaptic responses as seen in 5-HT favors slower rates of fictive locomotion (Kozlov et al., 2001; Svensson et al., 2001). Therefore, the complement of excitation of different glutamate receptors on VHN dendrites and the subsequent integration of those inputs, in conjunction with K_Ca_2 channels, is subject to serotonergic modulation of both synaptic transmission and intrinsic membrane properties. These very different effects of modulators impacting synaptic function and K_Ca_2 converge to influence the output of single neurons that scale to alter motor output.

Thus, the serotonergic system in the spinal cord plays a crucial role in modulating the output of the spinal network. While these results, whether mediated by pre- (Schwartz et al., 2005; Gerachshenko et al., 2009) or postsynaptic (Harris-Warrick and Cohen, 1985; Wallén et al., 1989; Wikström et al., 1995) 5-HT receptors explain effects of exogenous 5-HT, pharmacological application obscures crucial information regarding the spatiotemporal pattern of 5-HT release during swimming. Nevertheless, it is clear that 5-HT has profound effects on neural patterns and phase relationships within the spinal cord during locomotion and that this effect is substantially mediated through effects on K_Ca_2 channel activation.

## Importance of studying dendritic properties within a behaving network

In all vertebrates, 5-HT and glutamate applied exogenously can initiate and influence locomotor-like activity. While it is remarkable that systemic drug application can reliably produce ethologically relevant locomotor patterns in lamprey (Sigvardt et al., 1985) and in other model systems (Rossignol et al., 1998; Kyriakatos et al., 2011), NMDARs *in vivo* are not physiologically activated by a tonic and diffuse release of glutamate. Instead, the release of neurotransmitter and subsequent receptor binding is exquisitely targeted to discrete postsynaptic loci with temporal precision. The physiological activation of NMDARs in any circuit is almost entirely mediated by the synaptic release of glutamate. This will only occur at synapses, and only following presynaptic release of glutamate at those synapses. This constrains the activation of NMDARs spatially and temporally, as well as the K_Ca_2 channels that are subsequently activated.

While pharmacological activation of the spinal network is presumably far from physiological, it has remained to be demonstrated just how distinct this artificial induction is from supraspinal control of descending command neurons and subsequent spinal CPG activation. It is important to note that generating rhythmic activity and appropriate phase coupling has many theoretical solutions (Wallén et al., 1992; Williams, 1992). In the spinal network that generates swimming, there can be multiple pathways which achieve a similar behavioral mode (Menelaou and McLean, 2012), an idea that emerged from the study of invertebrate CPGs (Marder and Bucher, 2007). In *Xenopus* larval tadpoles, there may be little specificity in anatomical connections early in development (Li et al., 2007) suggesting that precise dendritic targeting is not necessary for functional circuit formation. Instead, a very basic scaffolding of neuronal connections is sufficient to construct early behaviors (Roberts et al., 2014). However, the specificity of microcircuit connectivity is subject to change. Synapses are plastic as is the dendritic architecture (Kishore and Fetcho, 2013). Nevertheless, synaptic connectivity and subsequent location-dependent dendritic integration is paramount to neural computation within microcircuits controlling behavior.

Furthermore, our understanding of how monoamines in general and 5-HT in particular act *in vivo* is even less certain than glutamate because exogenous application of these modulators over an artificially and pharmacologically activated network merely compounds errors and cannot match physiological release. Indeed, monoamine cocktails with glutamate agonists evoke spinal network activity (Rossignol et al., 1998; Masino et al., 2012) and when applied individually to active networks, monoamines modulate network activity (Barbeau and Rossignol, 1990; Rossignol et al., 1998). To develop a comprehensive understanding of the true pattern of synaptic drive to spinal neurons and microcircuits requires a more physiological method of activation of these spinal circuits than has previously been employed (Issberner and Sillar, 2007; Dubuc et al., 2008; McLean et al., 2008; Kyriakatos et al., 2011), while retaining the capacity to study them directly from the subcellular to systems level.

Bath-applied NMDA leads to a large increase in baseline Ca^2+^_i_ while Ca^2+^ oscillations are synchronized throughout the dendrites of a single neuron (Bacskai et al., 1995; Viana di Prisco and Alford, 2004). In this context, all NMDARs will become active and are independent of presynaptic release of glutamate. Under these conditions, the precise relationship between activated routes of Ca^2+^ entry, whether NMDARs or VGCCs, may become obscured. This may allow K_Ca_2 channels to couple to Ca^2+^ microdomains as opposed to nanodomains implied by their physiological BAPTA sensitivity. Indeed, in VHNs dialyzed with EGTA, oscillation plateau progressively lengthen immediately following whole-cell access, on a time course equivalent to the diffusion of dyes to the most distal dendrites (Alpert and Alford, 2013). This may be interpreted as a progressive increase in EGTA-mediated Ca^2+^ buffering into the distal dendrites where Ca^2+^ oscillations are largest (Viana di Prisco and Alford, 2004), preventing some Ca^2+^ from binding K_Ca_2 channels to cause the repolarization (Alpert and Alford, 2013). This effect suggests that Ca^2+^ diffusing greater distances from its site of entry can, under certain circumstances, activate K_Ca_2 channels, which then contribute to the repolarization. Nevertheless, even under these non-physiological conditions of bath applied NMDA, cells dialyzed with BAPTA displayed immediate, severely impaired repolarization (Alpert and Alford, 2013).

The ability of NMDAR-induced Ca^2+^ entry to bind K_Ca_2 channels in Ca^2+^ microdomains may be an artifact of bath-applied NMDA and the robust increase in intracellular Ca^2+^, which may also cause Ca^2+^-induced Ca^2+^ release from internal stores. However, it was recently demonstrated that this K_Ca_2 channel conductance is physiologically activated by synaptically driven NMDAR-mediated Ca^2+^ entry (Alpert and Alford, 2013) and is vital for the proper functioning of the network during brain-evoked locomotion (Nanou et al., 2013). If the spatially and temporally precise synaptic activation of glutamate receptors during locomotion is sensitive to blockade of K_Ca_2 channels, then it should follow that there is complementary patterning dendritic Ca^2+^ entry which drives the activation of K_Ca_2 channels important for rhythm generation. However, we know very little about the spatiotemporal pattern of dendrite activation during behavior and how synaptic input is integrated in real time to impact cell output.

Several recent advances have made it possible to begin to assess how dendrites integrate incoming synaptic information within an active, behaving network. Dendritic spatiotemporal Ca^2+^ dynamics in active networks are crucial to understanding how physiological patterns of synaptic input are integrated in real time to shape the cellular output and have only recently been investigated. With new advances in genetically encoded Ca^2+^ indicators (Muto et al., 2011) and *in vivo* 2-photon microscopy, it is now becoming possible to “watch dendrites in action” and correlate their activity to sensory input and behavioral output (Dombeck et al., 2010; Xu et al., 2012; Smith et al., 2013b; Grienberger et al., 2014). However, particularly in the lamprey model system, but presumably in other systems like zebrafish, there is a distinct advantage in imaging dendritic behavior-the activity of spinal motoneuron and interneuronal dendrites and the subsequent electrical output of individual cells can be precisely correlated to the real time network output, whose role in generating behavior is well characterized and directly measureable. Such multilevel analyses will undoubtedly enhance our understanding of how nervous systems generate behavior from subcellular to systems level with unprecedented detail.

## Conflict of interest statement

The authors declare that the research was conducted in the absence of any commercial or financial relationships that could be construed as a potential conflict of interest.
